# The Portuguese Severe Asthma Registry: Development, Features, and Data Sharing Policies

**DOI:** 10.1155/2018/1495039

**Published:** 2018-11-21

**Authors:** Ana Sá-Sousa, João Almeida Fonseca, Ana Margarida Pereira, Ana Ferreira, Ana Arrobas, Ana Mendes, Marta Drummond, Wanda Videira, Tiago Costa, Pedro Farinha, José Soares, Pedro Rocha, Ana Todo-Bom, Anna Sokolova, António Costa, Beatriz Fernandes, Carla Chaves Loureiro, Cecília Longo, Cecília Pardal, Célia Costa, Cíntia Cruz, Cláudia Chaves Loureiro, Cristina Lopes, Duarte Mesquita, Emília Faria, Eunice Magalhães, Fernando Menezes, Filipa Todo-Bom, Francisca Carvalho, Frederico S. Regateiro, Helena Falcão, Ivone Fernandes, João Gaspar-Marques, Jorge Viana, José Ferreira, José Manuel Silva, Laura Simão, Leonor Almeida, Lígia Fernandes, Lurdes Ferreira, Mafalda van Zeller, Márcia Quaresma, Margarida Castanho, Natália André, Nuno Cortesão, Paula Leiria-Pinto, Paula Pinto, Paula Rosa, Pedro Carreiro-Martins, Rita Gerardo, Rui Silva, Susana Lucas, Teresa Almeida, Teresa Calvo

**Affiliations:** ^1^Center for Health Technology and Services Research (CINTESIS), Faculdade de Medicina, Universidade do Porto, Porto, Portugal; ^2^Department of Community Medicine, Information, and Health Sciences (MEDCIDS), Faculdade de Medicina Universidade do Porto, Porto, Portugal; ^3^Allergy Unit, Instituto & Hospital CUF Porto, Porto, Portugal; ^4^Pulmonology Department, Centro Hospitalar e Universitário de Coimbra, Coimbra, Portugal; ^5^Immunology and Allergy Department, Centro Hospitalar Lisboa Norte, EPE, Lisboa, Portugal; ^6^Pulmonology Department, Centro Hospitalar de S. João, EPE, Porto, Portugal; ^7^Pulmonology Department, Faculty of Medicine University of Porto, Porto, Portugal; ^8^I3S Instituto de Investigação e Inovação em Saúde, Universidade do Porto, Porto, Portugal; ^9^Pulmonology Department, Centro Hospitalar Lisboa Norte, EPE, Lisboa, Portugal; ^10^VirtualCare, Porto, Portugal; ^11^Immunoallergology Department, Centro Hospitalar e Universitário de Coimbra, Coimbra, Portugal; ^12^Immunoallergology Department, Faculdade de Medicina, Universidade do Coimbra, Coimbra, Portugal; ^13^Immunology and Allergy Department, Hospital Prof. Doutor Fernando Fonseca, EPE, Amadora, Portugal; ^14^Pulmonology Department, Hospital da Senhora da Oliveira, Guimarães EPE, Guimarães, Portugal; ^15^Pulmonology Department, Hospital de Braga, Braga, Portugal; ^16^Department of Pediatrics, Centro Hospitalar e Universitário de Coimbra, Coimbra, Portugal; ^17^Pulmonology Department, Hospital Prof. Doutor Fernando Fonseca, EPE, Amadora, Portugal; ^18^Immunology and Allergy Department, Centro Hospital de Setúbal, EPE, Setúbal, Portugal; ^19^Pulmonology Unit, Hospitais da Universidade de Coimbra, Centro Hospitalar e Universitário de Coimbra, Portugal; ^20^Centre of Pulmonology, Faculty of Medicine, University of Coimbra, Coimbra, Portugal; ^21^Immunology and Allergy Department, Hospital Pedro Hispano Unidade Local de Saúde Matosinhos, EPE, Matosinhos, Portugal; ^22^Immunology Dpeartment, Faculdade de Medicina da Universidade do Porto, Porto, Portugal; ^23^Novartis Farma-Produtos Farmacêuticos, S.A., Porto Salvo, Portugal; ^24^Immunology and Allergy Department, Centro Hospitalar e Universitário de Coimbra, Coimbra, Portugal; ^25^Pulmonology Department, Centro Hospitalar Cova da Beira, EPE, Covilhã, Portugal; ^26^Pulmonology Department, Hospital Garcia de Orta, EPE, Almada, Portugal; ^27^Pulmonology Department, Hospital Beatriz Ângelo, Loures, Portugal; ^28^Immunology and Allergy Department, Centro Hospitalar do Porto, EPE, Porto, Portugal; ^29^Pulmonology Department, Centro Hospital de Setúbal, EPE, Setúbal, Portugal; ^30^Immunology and Allergy Department, Centro Hospitalar de Lisboa Central, EPE, Lisboa, Portugal; ^31^CEDOC, Integrated Pathophysiological Mechanisms Research Group, Lisboa, Portugal; ^32^Immunology and Allergy Department, Centro Hospitalar de Vila Nova de Gaia/Espinho, EPE, Vila Nova de Gaia, Portugal; ^33^Pulmonology Department, Unidade Local de Saúde da Guarda, EPE, Guarda, Portugal; ^34^Pulmonology Department, Centro Hospitalar Tâmega e Sousa, EPE, Penafiel, Portugal; ^35^Pulmonology Department, Hospital Distrital Figueira da Foz, EPE, Figueira da Foz, Portugal; ^36^Pulmonology Department, Faculdade de Medicina, Universidade do Porto, Porto, Portugal; ^37^Department of Pediatrics, Centro Hospitalar de Trás-os-Montes e Alto Douro, EPE, Vila Real, Portugal; ^38^Pulmonology Department, Centro Hospitalar do Oeste, Torres Vedras, Portugal; ^39^Pulmonology Department, Hospital da Luz Arrábida, Vila Nova de Gaia, Portugal; ^40^ISAMB, Instituto de Saúde Ambiental Faculdade de Medicina de Lisboa. Lisboa, Portugal; ^41^Pulmonology Department, Hospital de Vila Franca de Xira, Vila Franca de Xira, Portugal; ^42^Pulmonology Department, Centro Hospitalar de Lisboa Central, EPE, Lisboa, Portugal; ^43^Immunology and Allergy Department, Centro Hospitalar de Trás-os-Montes e Alto Douro, EPE, Vila Real, Portugal; ^44^Pulmonology Department, Centro Hospitalar Universitário do Algarve, Faro, Portugal; ^45^Pulmonology Department, Centro Hospitalar de Trás-os-Montes e Alto Douro, EPE, Vila Real, Portugal

## Abstract

The Portuguese Severe Asthma Registry (*Registo de Asma Grave Portugal,* RAG) was developed by an open collaborative network of asthma specialists. RAG collects data from adults and pediatric severe asthma patients that despite treatment optimization and adequate management of comorbidities require step 4/5 treatment according to GINA recommendations. In this paper, we describe the development and implementation of RAG, its features, and data sharing policies. The contents and structure of RAG were defined in a multistep consensus process. A pilot version was pretested and iteratively improved. The selection of data elements for RAG considered other severe asthma registries, aiming at characterizing the patient's clinical status whilst avoiding overloading the standard workflow of the clinical appointment. Features of RAG include automatic assessment of eligibility, easy data input, and exportable data in natural language that can be pasted directly in patients' electronic health record and security features to enable data sharing (among researchers and with other international databases) without compromising patients' confidentiality. RAG is a national web-based disease registry of severe asthma patients, available at* asmagrave.pt*. It allows prospective clinical data collection, promotes standardized care and collaborative clinical research, and may contribute to inform evidence-based healthcare policies for severe asthma.

## 1. Introduction

Severe asthma has been defined as asthma which requires treatment with high dose inhaled corticosteroids plus a second controller (and/or systemic corticosteroids), to prevent it from becoming “uncontrolled” or asthma which remains “uncontrolled” despite this therapy [1].

To improve care, a better understanding of the etiology, burden and management patterns of severe asthma is needed. The management of severe asthma is challenging and involves treatment of comorbidities, medication adherence, allergens exposure avoidance, among others. One of the greatest difficulties is the choice of the optimal treatment for each given patient, although algorithms for treatment decisions have been suggested [2, 3]. Monoclonal antibodies targeting immunoglobulin-E (IgE) and interleukin-5 are currently available and new biologics are under development. However, it is not easy to choose between the biologics to be the first-choice treatment, and head-to-head comparison studies between them do not exist [4]. A trial involving the direct comparison of two or more treatments is a pressing needed, but it may never be carried out [4]. Hence, clinical observational studies of real-world large patient populations should contribute to the knowledge on how to select the best biologic treatment for an individual patient.

Disease registries are recognized as powerful tools to improve disease-related knowledge. They consist of organized systems that use observational study methods to collect uniform data aiming at evaluating specific outcomes for a heterogeneous population defined by a particular disease [5]. This type of study design enables the assessment of the effect of different therapies in the context of a single disease. Severe asthma registries are being created throughout Europe including in the United Kingdom (UK), Belgium, Germany, Austria, Netherlands, Italy, and Spain (Table 1). However, research aiming at reducing the disease-related burden requires prospective long-lasting studies and the coordination of a wide range of expertise, often only available at an international or even global level [6]. With the goal of establishing a global collaborative initiative, the International Severe Asthma Registry was created and the enrollment of 10 national registries is expected by December 2018[7]. The European Respiratory Society (ERS) Research Agency promotes collaborative Europe-wide research based on data collected from disease registries [8]. Its actions include the development of Standard Operational Procedures and guidelines, consent forms to collect and handle data in compliance with the EU legal and regulatory framework, and establishing a central point to access datasets from multiple projects. In 2016 the collaboration Severe Heterogeneous Asthma Research collaboration, Patient-centered (SHARP) was accepted as an ERS Clinical Research Collaborations [9]. Taking this into consideration, new registries should be designed to enable sharing information and coordination among databases (e.g., federated databases).

Asthma affects 6.8% of the Portuguese population [10]. Using the data from the Portuguese National Asthma Survey we estimate 7.4% of patients were on step 4 or 5 treatment as defined by Global Initiative for Asthma (unpublished data). Even though severe asthma patients represent only a small proportion of those with asthma, they account for a large proportion of asthma-related morbidity and health care expenditures [11].

REAG,* Rede de Especialistas em Asma Grave*, is an open collaborative network of asthma specialists (allergists, pediatricians, and pulmonologists) who manage severe asthma patients in Portuguese hospitals. The foundational principle of REAG is the informal peer collaboration among colleagues with different medical specialties and backgrounds, maintaining an unhierarchical organization and consensual decision processes to improve sharing of medical experience, data, and knowledge. Since 2011, this network of experts has been working towards a better care of severe asthma patients by (1) promoting a better coordination between medical specialties for early diagnosis and referral of severe asthma patients; (2) describing and implementing harmonized procedures to adopt in severe asthma healthcare; and (3) improving scientific knowledge on severe asthma in Portugal. In 2015, REAG published a real-life prospective study on Portuguese patients with severe persistent allergic asthma, treated with omalizumab [12]. This was the first-time specialists from different Portuguese centers who made an effort to harmonize the registration procedures for severe asthma. From this initial study, the necessity for a computerized disease registry became even more evident.

The purpose of the Portuguese Severe Asthma Registry (*Registo de Asma Grave Portugal (RAG)*) is to gather evidence on severe asthma in Portugal contributing to eliminate the information gaps and support the adoption of evidence-based health care policies. Specifically, the registry aims at

1. improving the healthcare delivery of severe asthma by identifying the best diagnosis and treatment practices and by standardizing disease management processes and clinical records;
2. supporting collaborative research projects by promoting the cooperation between centers and assist with the implementation of research projects.

In this paper, we describe the development and implementation of RAG, its features, and data sharing policies.

## 2. Material and Methods

RAG results from the collaboration between different stakeholders: the medical experts from REAG, the investigators from CINTESIS (Center for Health Technology and Services Research), and the software development company VirtualCare.

**Figure 1:**
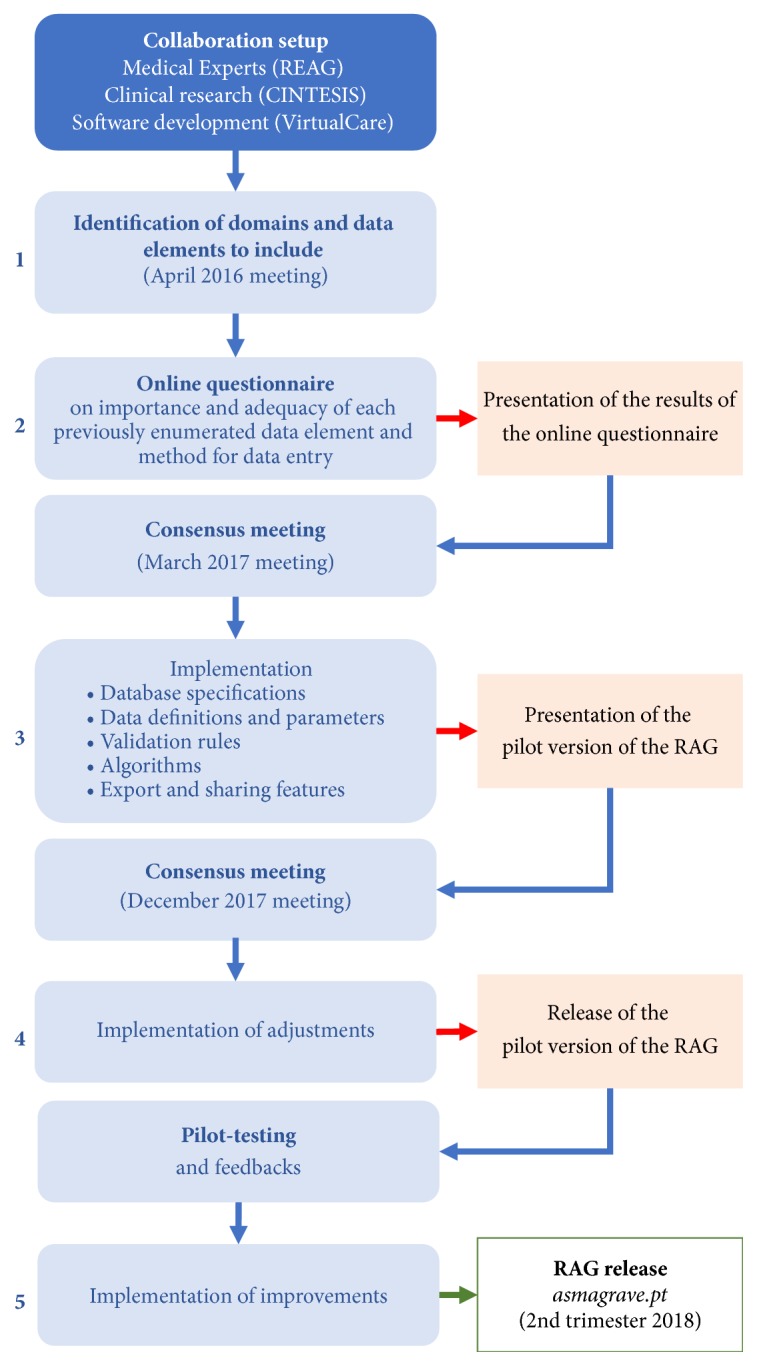
Development and implementation process of RAG.

The development and implementation processes of RAG are summarized in Figure 1.

### 2.1. Definition of Contents

The criteria for patient inclusion in RAG, the domains, and data elements to be registered were defined by a multistep consensus method.

The patients' inclusion criteria were based on the definition of Severe Asthma by GINA [1]: (1) patient with treatment on step 4 or 5 according to GINA recommendations; and (2) verified optimization of treatment adherence and comorbidities management. An additional inclusion criterion was (3) the patient's signed consent to have his/her data included in the registry.

During a meeting (April 2016), the domains and data elements were enumerated, based on the medical expertise of the network and taking into consideration the variables existing in three existing European Registries: the Belgian, the German, and the UK Severe Asthma Registries. Both data elements to be included in the initial patient registry and relevant follow-up information were identified. Different data entry methods were considered to reduce the burden of response.

An online questionnaire sent to 79 medical specialists from REAG was used to explore the importance of each data element and adequacy of data entry method. A total of 34 participants (43%) completed the questionnaire. For each domain, data elements and methods for data entry were chosen when gathering at least 80% of the votes. Comments and suggestions regarding additional variables or different data entry methods were also considered. The results of the questionnaire were presented in a meeting (March 2017) and disagreements were solved by consensus.

### 2.2. Features

Database specifications concerning data definitions and parameters and data validation rules were determined. To assist confirmation of the first criterion and support decision-making, an algorithm to automatically determine the step of treatment based on currently used asthma medication was created.

The following additional features were implemented:

- Support on data entry by automatic validation of the inserted data and error messages
- Creation of automatic reports, based on the information stored, to be integrated into the institutional electronic health record (the data recorded are exportable in natural language by generating a text that mimics clinical notes)
- Graphic display of aggregated data on patients' inclusion by healthcare center
- Display for each physician a list of their patients and date of the last medical appointment
- At follow-up visit, automatic display of the information inserted in the last appointment for specified measurements
- Export features for potential data exchange with international severe asthma databases and the pharmacovigilance authorities
- Automatic emails with status report of each registration

### 2.3. Security and Data Sharing Policies

Security features compliant with the new European General Data Protection Regulation (GDPR) [37] and required procedures according to this legislation are being incorporated into the platform.

The registry was built on a framework residing in a server hosted by VirtualCare. This server was configured with a Secure Sockets Layer (SSL) certificate from Comodo Security Solutions, Inc., ensuring that all data transferred between the web server and browsers remain private and integral. The access to the database is restricted, requiring authentication (using health professional number and password) and all accesses to the database are stored and traceable. All changes to the database are also stored; each change generates a new document; the old document becomes out of date allowing the tracking of changes (when, where, and by whom changes were made to the documents).

RAG does not record any identifiable personal data from patients (e.g., date of birth is replaced by the year of birth, no ID numbers are registered, and patients' names are pseudoanonymized so replaced with a code number) [38]. The patients' participation on RAG is free and voluntary, and patients may, in any moment and without penalty, withdraw the registry or verify and/or delete their data, by contacting the technical support. Patients are informed on the purposes of RAG, the data collected, and the implications of participating in this registry. The informed consent form is automatically generated at the time of inclusion. Only patients that agree, by a clear affirmative consent given by a written statement, to the storage, processing, and sharing of data belonging to him/her are included in RAG. The signed consent forms are upload into the application server file system, encrypted using phpseclib's library of PHP, which allows the usage of one of its encryption algorithms combined with a private key. When encrypted, the consent file cannot be read unless the file decryption is activated with the correct combination of algorithm and private key. The algorithm and private key are known only to VirtualCare.

An informed consent is also required by physicians who are registered in RAG since they provide identifiable personal data for that registration, namely, name, health professional number, and email address. At the time of registration, physicians must indicate their acceptance by ticking a box with a clear statement on the storage and processing of their personal data. The registration of each physician in RAG must be validated by at least one of five members of REAG, designated coordinators of RAG.

Data within RAG belongs primarily to each patient and then to the physician that inserted patients' data into the registry. Each physician is responsible for the management of the data that he/she inputted, belonging to his/her patients. Access to patients' data by their physicians is based on the Role Based Access Control (RBAC) model that associates privileges and permissions to the roles (e.g., professional categories). This model allows easier administration and independence in relation to the system users and permissions associated with its resources.

After authentication, each physician can access all the registrations inserted by himself/herself, both for clinical and research purposes. One local coordinator will be designated in each center. Each center coordinator has access, for pressing clinical purposes only, to all data inserted by the physicians in that center. If a patient changes the attending physician, the new physician, if interested in having access to the previously inserted data, must request authorization to the former physician, with patient's consent. Local and national coordinators and RAG technical support may assist this contact.

Data inserted by other physicians may be shared within REAG for research purposes, after authorization. For this, the physician proposing the data analysis must fill-in a form containing the aim and a brief description of the research project and the principal investigator or research group. When a request for abstracting data is filled, each physician with data matching the request is notified by email and has a period of 5 days to refuse the sharing of the data. In the case of shared information, the privacy of the individual is assured, as registry data cannot be individually identifiable.

### 2.4. Pilot-Test

After the implementation of the selected data elements, the supporting features, and validation rules, a beta version of RAG was presented during a REAG meeting (December 2017) and, after adjustments, it was pilot-tested for a month. The pilot version was tested by 22 REAG members and 85 specific feedback comments were provided by 8 testers. The first version of RAG became ready after improvements being made based on the pilot-test feedback.

## 3. Results

The Portuguese Severe Asthma Registry is a national web-based disease registry. The access is made from the website of REAG,* asmagrave.pt*, after authentication.

**Figure 2:**
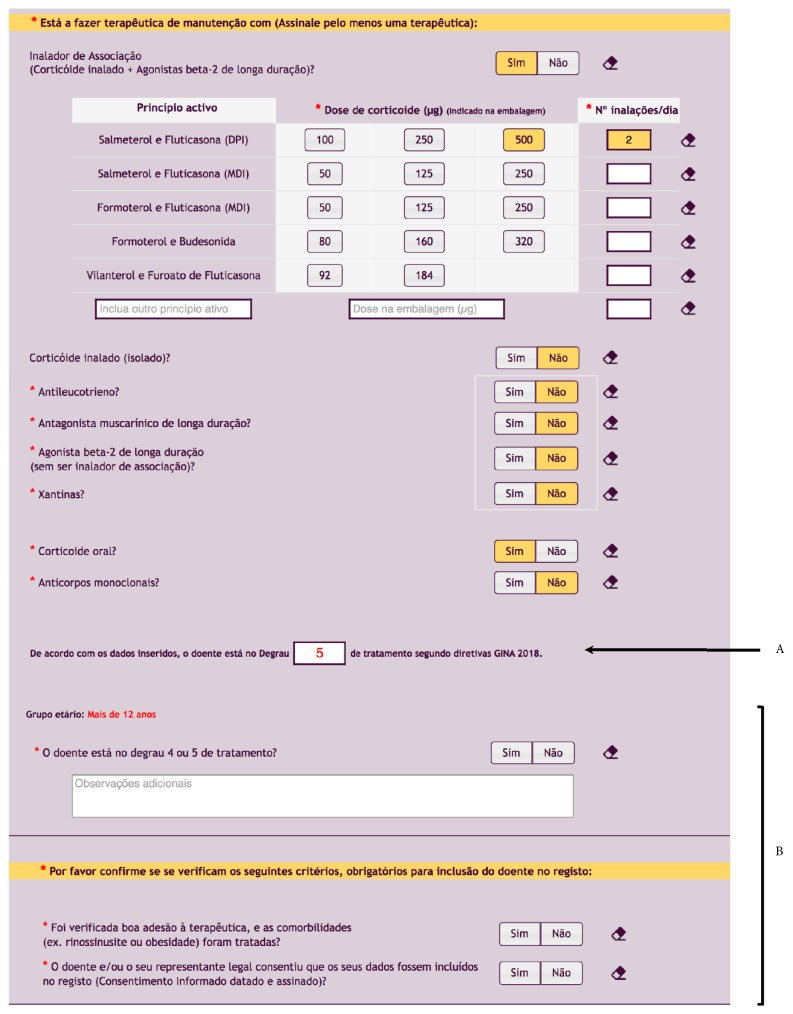
Screenshot of the implemented automatic algorithm to determine the step of treatment, based on asthma medication according to GINA recommendations. A: treatment step calculated by the algorithm; B: the 3 criteria for patients' inclusion.

RAG gathers data of adults and children with severe asthma followed at specialized care centers which, after treatment optimization and adequate management of comorbidities, require step 4 or 5 of treatment according to GINA recommendations[1]. The implemented automatic algorithm determines the step of treatment for patients aged under 6, between 6 and 12 and over 12 years, based on asthma medication prescribed to the patient according to GINA recommendations (Figure 2.A). In any case, the physician makes the decision about the inclusion in the registry indicating the reason for inclusion (Figure 2.B). In fact, even if rarely used, some therapeutic combinations are not explicitly considered in any of the GINA 2018 treatment steps and in these cases, the algorithm cannot present a result. The algorithm will be updated in the future when these recommendations change.

**Figure 3:**
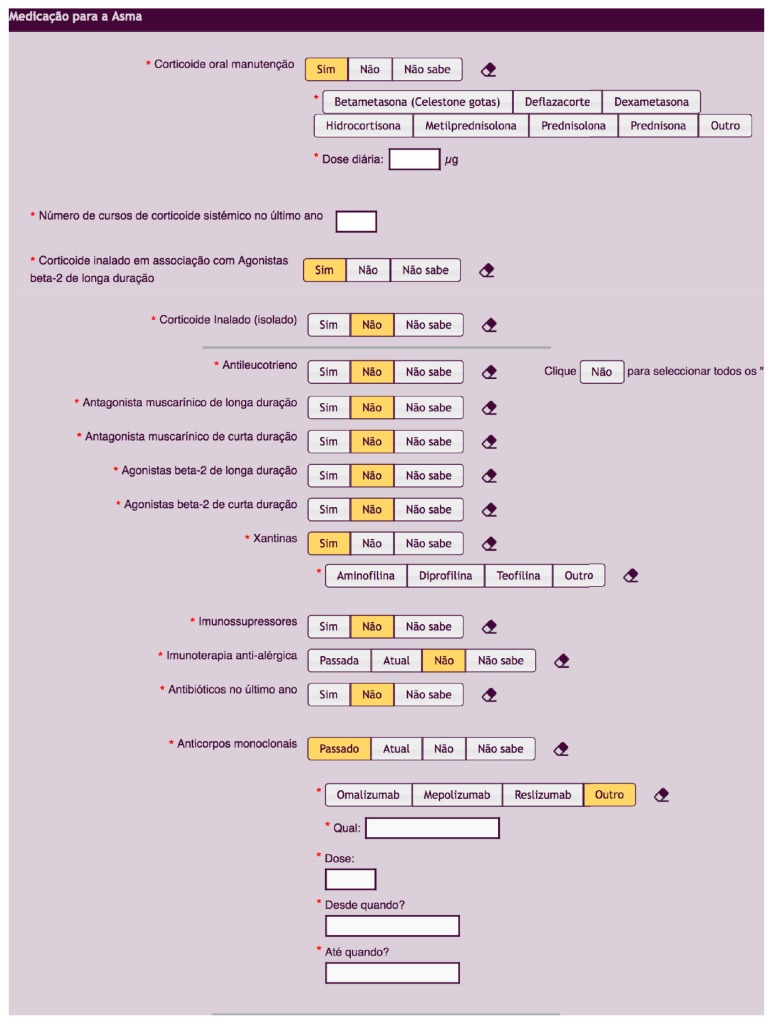
Screenshot of RAG, picturing asthma medication being collected by RAG.

The final data items of RAG are summarized in Table 2. RAG allows collecting data on different asthma medication, including Oral Corticosteroids (OCs), monoclonal antibodies, and even new therapies that may become available (Figure 3). Data considered as essential are compulsory, whereas desirable but not essential data may be skipped. The elements to be collected in the follow-up appointments were also defined as RAG was designed to collect data prospectively.

## 4. Discussion

The Portuguese Severe Asthma Registry is a national web-based disease registry of adult and pediatric severe asthma patients. It includes a comprehensive list of data elements defined by a multistep consensus process, supported by international definitions of severe asthma. The registry offers features to facilitate data entry and to support decision-making. The collected data belongs primarily to each patient and then to the physician who inserted patients' data into the registry and can be shared for research purposes after authorization. A thorough characterization of severe asthma patients, using a tool consensually defined to be applied prospectively by specialists from Portuguese hospitals, is ambitious but can improve the information on the disease and contribute to the adoption of evidence-based policies for severe asthma care. This harmonized approach is essential to improve the management of the different phenotypes this pathology. The Portuguese registry was designed to enable future linkage with other databases, as registries from other countries, as well as the Portuguese Pharmacovigilance Authority.

The data elements included in RAG were selected to reflect the current clinical status of the patient avoiding unnecessary burden within the clinical workflow. Through a multistep consensus method, a balance was achieved between the data commonly used by clinicians, the data included in other severe asthma registries, the data needed for the RAG's reliability, and the expected overall burden for respondents. Therefore, there was an effort to data collected by RAG which can be compared to data collected by other registries enabling comparisons across populations and settings. A consensus method was used to summarize information from different sources, to gather insights from experts and to enable decision-making [39]. After the selection and implementation of the data elements and validation rules, RAG was pilot-tested and iteratively improved before release.

The patients' inclusion criteria were also defined by consensus and an automatic algorithm was implemented to assist patients' eligibility assessment, based on GINA recommendations. Clinical guidelines provide a link between the best available evidence and the clinical practice, having the potential to improve enormously patient care [40]. However, these may have limitations especially for a particular disease where evidence is still insufficient as in severe asthma and cannot be used as a strict formula. During algorithm development became clear that GINA 2018 treatment steps do not account for all possible therapeutic combinations. In the future, it would be important to assess if clinically relevant combinations are not included in the GINA recommendations, to contribute to the improvement of the recommendations concerning severe asthma.

Disease registries are used to support healthcare providers on disease care and to gather evidence for scientific and policy purposes. Therefore, a disease registry should (1) facilitate the access to patient-specific information at the point of care for healthcare delivery and provide status reports of aggregated information to give feedback to physicians or to medical groups about the patient population [36] and (2) provide real-world data on clinical practice, patient outcomes, safety, and/or comparative effectiveness for research purposes[5]. RAG has several features to support healthcare providers on severe asthma care (Table 3). Additionally, as suggested by the members of REAG, RAG includes the automatic generation of clinical notes based on the inputted data that can be pasted into the institutional electronic clinical record of the patient, avoiding duplication of effort.

Real-world prospective observational research, including long-term follow-up data provided by registries, is increasingly considered important to generate evidence regarding effectiveness, safety, and quality of care [41]. The utility of a registry relies on the quality of data collection and storage [5]. RAG's data are collected at the time of routine medical appointments, in the same manner for every patient, with specific and consistent data definitions. To minimize errors related to data completeness and consistency, several logical and validation rules have been implemented and periodic data audits are being planned. An additional challenge is the recruitment and retention of participants that is critical to the generalizability of a registry [5]. Potential RAG users were involved from the beginning in the development and implementation process and stated their motivation to include patients. Nevertheless, to retain users' interest, the burden of participation was kept as low as possible and features wanted by the physicians were implemented.

RAG was designed to comply with security and data protection standards, including key challenges of the new European GDPR. No individually identifiable information of the patient is recorded in the database. Only the his/her physician can link the recorded data to the patient that remains the owner of the data. RAG's data sharing policies allow the use of data for research, requiring the consent of the physician that recorded the data and a simple process to gather this consent was implemented.

## 5. Conclusions

The Portuguese Severe Asthma Registry is a national web-based disease registry of adult and pediatric severe asthma patients. The development and implementation of the RAG was a multistep consensus process. RAG includes automatic assessment of eligibility, easy data input, and features for exporting and sharing data. It allows prospective clinical data collection, promotes standardized clinical records, and creates a secure virtual setting for collaborative clinical research. RAG database is prepared for future data exchange with international databases. In the future, the analysis of RAG data may contribute to inform evidence-based healthcare policies for severe asthma.

## Figures and Tables

**Table 1:** European Registries of Severe Asthma, a noncomprehensive review.

Registry name	Country	Year of release	Promoting Society	Website	Patients included	No. of centers	Sources / published studies
United Kingdom Severe Asthma Registry	United Kingdom	2006	British Thoracic Society	https://www.brit-thoracic.org.uk/standards-of-care/ lung-disease-registries/	>500	8	[13–22]

Belgian Severe Asthma Registry	Belgium	2008	Belgische Vereniging voor Pneumologie / Société Belge de Pneumologie	http://www1.citobi.be/SAR/Welcome_en.act	>350	9	[23, 24]

Register Schweres Asthma	Germany	2011	German Asthma Net e.V.	http://www.german-asthma-net.de	>100	17	[25, 26]

Banco de Datos de Asma	Spain	<2012	Sociedad Española de Neumologia y CirurgiaToracica	https://www.separ.es/?q=node/71	>290	30	[27, 28]

Austrian Severe Asthma Net	Austria	2012	Austrian Severe Asthma Net (ASA-Net)	http://www.asa-net.at/register/	>80	16	[29]

Severe/Uncontrolled Asthma Registry	Italy	2014	Italian Severe Asthma Network (SANI).	http://www.sani-asma.org	>400	63	[30, 31]

Registry of Adult Patients with Severe asthma for Optimal Disease management	Netherlands	2016	Academisch Medisch Centrum (Prof. dr. E.H.D. Bel)	https://www.zonmw.nl/nl/ over-zonmw/innovatie-in-de-zorg/programmas/ project-detail/goed-gebruik-geneesmiddelen/registry-of- adult-patients-with-severe-asthma-for-optimal- disease-managementrapsodi/verslagen/	>20	3	[32]

Registo de Asma Grave Portugal	Portugal	2018	Rede de Especialistas em Asma Grave	https://www.asmagrave.pt/	Release planned for 2nd trimester of 2018	31	-

**Table 2:** Domains and data elements recorded in the Portuguese Severe Asthma Registry.

**Patient data **	

Demographic data	
*(gender∗, birth of month∗ and year∗, birthplace, place of residence∗, body mass index calculation∗, education years∗, smoking habits∗, occupation∗, family history of asthma∗ and of *	
*asthma-related death∗, personal history of respiratory infections during early childhood∗, environmental exposures)*	

Asthma care information	
*(age at asthma diagnosis∗, age at severe asthma classification∗, first year of specialized asthma follow-up, medical specialty of the attending physician∗*)	

**Comorbidities** *∗*§	

**Atopy and Inflammation biomarkers**	

Atopy	
*(total serum IgE∗, allergic sensitization∗, type(s) of diagnostic test used to confirm allergic sensitization∗*)	

Inflammation biomarkers* (FeNO, blood eosinophils, sputum eosinophils, sputum neutrophils)*	

**Diagnostic tests**	

Lung function tests	
*(FEV1∗, FVC∗, MEF, residual volume, specific airway resistance, carbon monoxide diffusion capacity, bronchial challenge test)*	

Imaging	
*(thorax X-ray∗, thorax CT scan∗, sinus CT scan, bronchial endoscopy, bone densitometry)*	

Arterial blood gases	

**Control and Quality of Life**	

Asthma-related healthcare utilization due to asthma in previous 12 months (or since the last appointment, when at follow-up visit)	
*(number of routine primary care medical appointments, routine hospital care medical appointments, non-scheduled medical appointments∗*§*, emergency service admissions∗*§,	
*hospitalizations∗*§*, intensive care unit admissions, need for mechanical ventilation, school or labor absenteeism)*	

Asthma control assessment according to GINA recommendations[1]	
*(frequency of daytime symptoms∗*§*, activity limitations due to asthma∗*§*, any night awakening due to asthma∗*§*, frequency of use of reliever medications for asthma∗*§*, respiratory function, *	
*number of exacerbations in last year/week∗*§)	

Asthma control self-questionnaires *(CARAT∗*§* and external link to ACT)*	

Quality of life self-assessment questionnaires	
*(external link to quality of life self-assessment questionnaires)*	

**Therapy**	

Asthma medication*∗*§	
*(OCs, ICs, LTRAs, LABAs, SABAs, LAMAs, SAMAs, xanthines, immunosuppressors, immunotherapy, monoclonal antibodies, antibiotics, therapy adherence, inhalation technique)*	

Other medication	
*(proton pump inhibitor, anti-depressive/anxiolytics, intranasal steroids, antihistamines, long-term oxygen therapy, non-invasive ventilation)*	

*∗*Compulsory data elements at initial visit; § compulsory data elements at follow-up.

IgE: immunoglobulin-E; FeNO: Fractional exhaled Nitric Oxide; FEV1: forced expiratory volume in the first second; FVC: forced vital capacity; MEF: midexpiratory flow; CT: computed tomography scan; CARAT: Control of Allergic Rhinitis and Asthma Test [33, 34] and ACT: Asthma Control Test [35]; OCs: Oral Corticosteroids; ICs: inhaled corticosteroids, LTRAs: Leukotriene Receptor Antagonist; LABA: Long-Acting Beta 2 Agonist; SABA: Short-Acting Beta Agonist; LAMA: Long-Acting Muscarinic Antagonist; SAMA: Short-Acting Muscarinic Antagonist.

**Table 3:** RAG features useful to support severe asthma management.

**Elements of chronic care management **[36]	**RAG features**	
	Current	Future
**Ensure regular follow-up**	Displays for each physician a list of their patients and date of the last medical appointment	Display a simple message with the counting the months since the last appointment and flag patients without medical review in more than 6 months

**Facilitate individual patient care planning**	For specified measurements, displays the information inserted in the last appointment and its progress over time	At the beginning of each follow-up appointments, a brief report of the previous appointment will be displayed

**Embed evidence-based guidelines into clinical practice**	has a decision support tool to identify patients treated in step 4 or 5 according to GINA recommendations	

**Monitor the performance of practice team **	Displays aggregated data on the number of patients included by each center	Aggregated real-time data with different graphic displays of trends on specified management and clinical outcomes will be produced, to give a feedback to physicians about the status of the care of their patients and/or healthcare center, towards delivering the recommended care for severe asthma.

## Data Availability

Data sharing is not applicable to this article.
